# 

**Published:** 1897-06

**Authors:** 


*The following donations have been received since the publication of the list in March:*

*May 31st, 1897.*

| | |
|---|---|
| American Public Health Association (1) ... ... | 3 volumes. |
| George M. Gould, M.D. (2) ... ... ... ... ... ... | 18 ,, |
| L. M. Griffiths (3) ... ... ... ... ... ... ... ... | 2 ,, |
| C. B. Keetley (4) ... ... ... ... ... ... ... ... | 2 ,, |
| Laryngological Society of London (5) ... ... | 1 volume. |
| Pathological Society of London (6) ... ... ... | 2 volumes. |
| Young J. Pentland (7) ... ... ... ... ... ... ... | 2 ,, |
| Royal Academy of Medicine in Ireland (8) ... | 1 volume. |
| R. Shingleton Smith, M.D. (9) ... ... ... ... | 3 volumes. |
| Smithsonian Institution (10) ... ... ... ... ... ... | 1 volume. |

Unbound periodicals have been received from Mr. C. B. Keetley.

### TWENTY-FOURTH LIST OF BOOKS.

The titles of books mentioned in previous lists are not repeated.

The figures in brackets refer to the names of the donors, and show by whom the volumes were presented. The books to which no such figures are attached have either been bought from the Library Fund or received through the *Journal*.

| | | |
|---|---|---|
| **Allbutt, T. C.** [Ed.] | *A System of Medicine.* Vol. II. ... ... ... ... ... ... | 1897 |
| **Anderson, J.** ... | *Medical Nursing.* (Ed. by Ethel F. Lamport) 3rd Ed. | 1897 |
| **Anderson, W.** ... | *The Deformities of the Fingers and Toes* ... ... ... | 1897 |
| **Bishop, S. S.** ... | *Diseases of the Ear, Nose, and Throat* ... ... ... ... | 1897 |
| **Black, D. C.** ... | *The Medical Environment* ... ... ... ... ... ... ... | [1896] |

**Bocquillon-Limousin, H.** *Formulaire des Médicaments nouveaux* [*avec Supplément pour* 1897] ... ... ... ... 7th Ed. 1896

**Butler, G. F.** ... *Materia Medica, Therapeutics and Pharmacology*... ... 1897

**Coutts, J. A.** ... *Infantile Syphilis* ... ... ... ... ... ... ... ... ... 1897

**Davidson, F.** ... *Sight Testing for the G. P.* ... ... ... ... ... ... ... [N.D.]

**Elder, W.** ... ... *Aphasia and the Cerebral Speech Mechanism* ... ... 1897

**Fenwick, W. S.** ... *Disorders of Digestion in Infancy and Childhood*... ... 1897

**Finlayson, J.** ... *An Account of the Life and Works of Dr. Robert Watt* 1897

**Fitz, H. C. Wood and R. H.** *The Practice of Medicine* ... ... ... ... ... ... 1897

**Gray, W.** ... ... *Influenza* ... ... ... ... ... ... ... ... ... ... ... ... 1897

**Hall, A. J.** ... ... *Physiology—Students' Note-Book.* Part I.: *Physiological Chemistry* ... ... ... ... ... ... ... ... 1897

**Hawke, G. Pitt-Lewis, R. P. Smith and J. A.** *The Insane and the Law* ... 1895

**Hill, L.** ... ... *The Cerebral Circulation*... ... ... ... ... ... ... ... 1896

**Hughes, Amy** ... *District Nursing* ... ... ... ... ... ... ... ... ... 1897

**Jaeger, G.** ... ... *Problems of Nature.* (Ed. and Tr. by H. G. Schlichter) 1897

**Johnson, Theodora** *The Swedish System of Physical Education* ... ... ... 1897

**Knauer, O.** ... ... *Über Puerperale Psychosen* ... ... ... ... ... ... ... 1897

**Macnaughton-Jones, H.** *Gynæcology in Berlin* ... ... ... ... ... ... ... 1897

**McNeill, R.**... ... *Epidemics and Isolation Hospitals*... ... ... ... ... ... 1894

**Marcet, W.** ... ... *On the History of the Respiration of Man* ... ... ... 1897

**Moure, E. J.** ... *Traitement de l'Ozène. Rhinite atrophique fétide* ... 1897

**Moyes, J.** ... ... *Medicine in Shakespeare* ... ... ... ... ... ... ... ... 1896

**Orme, Miss S. E.**... *Hospital and Private Nursing* ... ... ... ... ... ... 1897

**Penna, J.** ... ... *El Cólera en la República Argentina* ... ... ... ... 1897

**[Phillips, F.]** ... *Moods: their mental and physical character* ... ... ... 1897

**Pitt-Lewis, R. P. Smith and J. A. Hawke, G.** *The Insane and the Law* ... 1895

**Pollack, B.** ... ... *Die Färbetechnik des Nervensystems* ... ... ... ... 1897

**Reynolds, Sir J. R.** *Essays and Addresses* ... ... ... ... ... ... ... ... 1896

**Richmond, C. E.**... *Antiseptic Principles for Nurses* ... ... ... ... ... 1897

**Roquete, J. I.** ... *Guia da Conversação Portuguez-Inglez* ... ... ... (3) 1843

**Senn, N.** ... ... *The Pathology and Surgical Treatment of Tumors* ... 1895

**Sforzosi, L.** ... ... *Conversations françaises et italiennes* ... (3) 2e Éd. 1837

**Simpson, Eve B.**... *Life of Sir J. Y. Simpson* ... ... ... ... ... ... ... [1896]

**Smith and J. A. Hawke, G. Pitt-Lewis, R. P.** *The Insane and the Law* ... 1895

**Squire, B.** ... ... *The Treatment of Lupus*... ... ... ... ... ... ... ... 1897

**Sutton, J. B.** ... *Ligaments: their nature and morphology* ... 2nd Ed. 1897

**Swanzy, H. R.** ... *Diseases of the Eye* ... ... ... ... ... ... ... 6th Ed. 1897

**Tirard, N.** ... ... *Diphtheria and Antitoxin* ... ... ... ... ... ... ... 1897

**Waring, H. J.** ... *Diseases of the Liver, Gall Bladder, etc.* ... ... ... 1897

**Watson, D. C.** ... *Diseases of the Eye* ... ... ... ... ... ... ... ... ... 1897

**Wood and R. H. Fitz, H. C.** *The Practice of Medicine*... ... ... ... ... 1897

---

### TRANSACTIONS, REPORTS, JOURNALS, &c.

Advertiser's A B C, The ... ... ... ... ... ... ... ... ... ... ... ... (9) [1890]
Alienist and Neurologist, The... ... ... ... ... ... ... ... Vol. XVII. 1896
American Journal of Obstetrics, The ... ... ... ... ... ...Vol. XXXIV. 1896

American Journal of Ophthalmology, The ... ... ... ... Vol. XIII. 1896
American Journal of the Medical Sciences, The Vols. I.—V., 1827–29; XI.—XX., 1832–37: N.S., Vols. XIII., XIV., 1847; XXIX., XXX. 1855
American Laryngological Association, Transactions of the, 1896 ... ... 1897
American Practitioner and News, The... ... ... ...Vols. XXI., XXII. 1896
American Public Health Association, Reports and Papers of the Vols. (1) XVIII.—XX., 1893–95; XXI. 1896
American Surgical Association, Transactions of the... ... Vol. XIV. 1896
American Year-Book of Medicine and Surgery, The .. ... ... ... 1897
Annali di Ostetricia e Ginecologia... ... ... ... ... ... ... .. ... 1896
Annals of Surgery ... ... ... ... ... ... ...(4) Vols. VI., VII. 1887–88
Archives cliniques de Bordeaux ... ... ... ... ... ... ...Tome V. 1896
Archives of Pediatrics ... ... ... ... ... ... ... ... Vol. XIII. 1896
Archives of Surgery ... ... ... ... ... ... ... ... .. Vol. VII. 1896
Archivio di Ortopedia ... ... ... ... ... ... ... .. ... ... 1896
Australasian Medical Gazette, The .. ... ... ... ... ...Vol. XV. 1896
Bristol Medico-Chirurgical Journal, The ... ... ... ... Vol. XIV. 1896
Bristol Port Sanitary District—Annual Report of the Medical Officers of Health for 1896... ... ... ... ... ... ... ... ... ... ... 1897
Bulletin de l' Académie de Médicine ... ... Tomes XXXV., XXXVI. 1896
Bulletin de l' Académie royale de Médecine de Belgique 4e Sér., Tome X. 1896
Canadian Practitioner, The ... ... ... ... ... ... ... Vol. XXI. 1896
Clinical Journal, The ... ... ... ... ... ... ... ... ...Vol. IX. 1897
Edinburgh Obstetrical Society, The Transactions of the Vols. XX., XXI. 1895–96
Giornale internazionale delle Scienze mediche ... ... ... ... ... ... 1896
Hospital, The ... ... ... ... ... ... ... ... ... ... Vol. XXI. 1897
Hospitals—
Saint Thomas's Hospital Reports ... ... ...N.S., Vol. XXIV. 1897
Westminster Hospital Reports, The... ... ... ... ... Vol. X. 1897
Intercolonial Medical Journal of Australasia ... ... ... ... Vol. I. 1896
International Medical Magazine ... ... ... ... ... ... ... Vol. V. 1897
Johns Hopkins Hospital Bulletin, The... ... ... ... ... ...Vol. VII. 1896
Journal of Nervous and Mental Disease, The ... ... N.S., Vol. XXI. 1896
Kentucky State Medical Society, Transactions of the ...N.S., Vol. V. 1896
Laryngological Society of London, Proceedings of the ... (5) Vol. III. 1897
Laryngoscope, The ... ... ... ... ... ... ... ... ... ... Vol. I. 1896
Library Year Book, Greenwood's ... ... ... ... ... . ... ... ... 1897
Liverpool Medico-Chirurgical Journal, The ... ... ... Vol. XVI. 1896
London and Edinburgh Monthly Journal of Medical Science, The (7) Vols. III., IV. 1843–44
Medical Chronicle, The ... ... ... ... ... ... ... N.S., Vol. VI. 1897
Medical News, The (2) Vols. XXXI.—XXXVII., 1873–79, XLII.—LV. 1883–89
Occidental Medical Times ... ... ... ... ... ... ... ... Vol. X. 1896
Pathological Society of London, Transactions of the (6) Vols. XLVI., XLVII. 1895–96
Philadelphia, Transactions of the College of Physicians of 3rd Ser., Vol. XVIII. 1896

Polyclinic, The ... ... ... ... ... ... ... ... (2) Vols. I.—VI. 1884–89
Post-Graduate, The ... ... ... ... ... ... ... ... ... Vol. XI. [1896]
Revue générale d' Ophtalmologie ... ... ... ... ... ... Tome XV. 1896
Revue médico-chirurgicale des Maladies des Femmes Tomes XVII., XVIII. 1895–96
Revue obstétricale et gynécologique ... ... ... ... ... Vol. XII. 1896
Royal Academy of Medicine in Ireland, Transactions of the (8) Vol. XIV. 1896
St. Louis Medical and Surgical Journal, The ... ... ... Vol. LXXI. 1896
St. Petersburger medicinische Wochenschrift ... ... ... ... ... ... 1896
Sanitary Record, The ... ... ... ... ... ... ... ... ... Vol. XVIII. 1896
Smithsonian Institution, Publications of the ... ... ... ... ... (10) 1896
Therapeutic Gazette, The ... ... ... ... ... ... ... ... Vol. XX. 1896
Wiener klinische Wochenschrift ... ... ... ... ... ... ... ... ... 1896
Year-Book of Scientific and Learned Societies ... ... ... ... ... ... 1897
Zeitschrift für Krankenpflege ... ... ... ... ... ... ... ... ... ... 1896

### Local Medical Notes.

### BRISTOL.

The twenty-fifth annual report of the Medical Missionary Society, being the one for the year 1896, records that 8074 patients were attended, an increase of 541 on the preceding year, and that every effort was taken to exclude persons who would not come under the heading of "sick poor." In fact the authorities seem keenly alive to the question of hospital abuse, to judge from the notices placed in various portions of the institution. The society appears to receive considerable support, and was fortunate in receiving a handsome legacy of £1000, though we regret to see that a sum is owing to the bankers. We wish Dr. Elder, medical superintendent, and his fellow-workers in the society success in the excellent medical and Christian work in which they are engaged.

University College.—On May 28th occurred the death of Mr. J. Greig Smith, the Professor of Surgery. A full notice of his life and work is given at the beginning of this number.

J. J. S. Lucas, B.A. Lond., M.R.C.S. Eng., L.R.C.P. Lond., has been appointed Medical Tutor in place of Dr. C. A. Brough, resigned.

The following students have recently passed examinations:

**Examining Board in England by the Royal Colleges of Physicians and Surgeons.**—*Five Years' Curriculum. First Examination. Part I. Chemistry and Physics*—P. L. Hickes, F. H. Rudge, R. H. N. Rutherford; *Part II. Practical Pharmacy*—F. P. Mackie; *Part III. Elementary Biology*—E. J. Dermott, P. L. Hickes, R. H. N. Rutherford. *Second Examination. Anatomy and Physiology*—A. Coleridge, F. W. Cotton, C. A. H. Gee, C. F. Walters. *Final Examination. Medicine*—J. J. S. Lucas*; *Midwifery*—C. D. Ingle, H. R. C. Newman.

* Completes Examination.

Bristol Royal Infirmary.—Mr. G. Munro Smith, M.R.C.S. Eng., Assistant-Surgeon, has been elected Surgeon. Mr. Thomas Carwardine, M.B., M.S., Lond., F.R.C.S. Eng., House-Surgeon, has been elected Assistant-Surgeon.

### PUBLICATIONS RECEIVED.

From PIETRO AGNELLI, Milan:

*Giornale della Reale Società Italiana d'Igiene.* 28 Febbraio, 15, 31 Marzo, 30 Aprile, 1897.

From ASH & Co., London:

*Hand-Book of the Clinical Research Association, Limited, with the Annual Report for* 1896.

From J.-B. BAILLIÈRE ET FILS, Paris:

*Formulaire des Médicaments nouveaux,* 7e Édition, avec Supplément pour 1897. Par H. Bocquillon-Limousin.

From BAILLIÈRE, TINDALL AND COX, London:

*The Analyst.* Vol. XXII. No. 254. May, 1897.

From JOHN BALE SONS & DANIELSSON, LIMITED., London:

*Gynæcology in Berlin.* By H. MACNAUGHTON-JONES, M.D., M.Ch., M.A.O. 1897.
*The Journal of Balneology and Climatology.* April, 1897.

From FRIEDR. BAYER & Co., Elberfeld:

*Clinical Excerpts.* Vol. I. Nos. 3—5. March, April, 1897.

From BENNETT BROTHERS, LIMITED, Bristol:

*Bristol Port Sanitary District. Annual Report of the Medical Officers of Health and of the Chief Port Inspector of Nuisances for the year* 1896. 1897.

From CALIFORNIA MEDICAL COLLEGE, San Francisco:

*California Medical Journal.* March, April, 1897.

From CASSELL AND COMPANY, LIMITED, London:

*Surgical Diseases of Children.* By EDMUND OWEN, M.B. Third Edition. 1897.

From J. & A. CHURCHILL, London:

*Antiseptic Principles for Nurses.* By C. E. RICHMOND. 1897.
*The Treatment of Lupus.* By BALMANNO SQUIRE, M.B. 1897.
*The Deformities of the Fingers and Toes.* By WILLIAM ANDERSON. 1897.
*The Cerebral Circulation.* By LEONARD HILL, M.B. 1896.

From CHURCH MISSIONARY SOCIETY, London:

*Mercy and Truth.* Vol. I. Nos. 4—6. April—June, 1897.

From WILLIAM F. CLAY, Edinburgh:

*Diseases of the Eye.* By D. CHALMERS WATSON, M.B., C.M. 1897.

From T. AND A. CONSTABLE, Edinburgh:

*Atlas of Clinical Medicine.* By BYROM BRAMWELL, M.D. Vol. III. In Three Parts. 1894—7.

From THE F. A. DAVIS COMPANY, Philadelphia:

*Diseases of the Ear, Nose, and Throat.* By SETH SCOTT BISHOP, M.D., LL.D. 1897.

From DESBARATS & Co., Montreal:

*Preliminary Programme. British Medical Association.* 65th Annual Meeting. Aug. 31 to Sept. 4, 1897.

From O. H. DREYER, St. Louis:

*Medical Review.* February 27, March 6, 20, 27, April 3, 10, 17, 24, May 1, 8, 15, 22, 29, 1897.

From EDUARDO DUBLAN, Mexico:

*Boletin del Consejo superior de Salubridad.* Enero, Febrero, 1897.

From G. DUPONT, Grenoble:

*Uriage (Isère): its Site, its Springs, its Baths.* 1897.

From FERET ET FILS, Bordeaux:

*Traitement de l'Ozène.* Par le Dr. E. J. MOURE. 1897.

*De l'Ouverture large de la Caisse et de ses Annexes.* Par le Dr. E. J. MOURE. 1897.

From Dr. L. WEBSTER FOX, Philadelphia:

*Ulcers of the Cornea—Implantation of a Glass Ball for the Better Support of an Artificial Eye.* By L. WEBSTER FOX, M.D. 1896. [Reprint.]
*Introductory Clinical Lecture.* By L. WEBSTER FOX, M.D. 1896. [Reprint.]
*Ophthalmia Neonatorum.* By L. WEBSTER FOX, M.D. 1897. [Reprint.]

From EUGEN GROSSER, Berlin:

*The Alkalies, Lime in particular, as Factors in the Treatment for the Elimination of Uric Acid.* By LIVIUS FÜRST, M.D. [N.D.]

From INGRAM BROTHERS, London:

*The English Illustrated Magazine.* April—June, 1897.

From S. KARGER, Berlin:

*Über Puerperale Psychosen.* Von Dr. OSWALD KNAUER. 1897.
*Die Färbetechnik des Nervensystems.* Von Dr. B. POLLACK. 1897.

From W. KUHLENTHAL, London:

*S—P—O—R Notes.* No. 1. December, 1896.

From H. K. LEWIS, London:

*Medical Nursing.* By the late JAMES ANDERSON, M.D. Edited by ETHEL F. LAMPORT. Third Edition. 1897.
*Aphasia and the Cerebral Speech Mechanism.* By WILLIAM ELDER, M.D. 1897.
*Disorders of Digestion in Infancy and Childhood.* By W. SOLTAU FENWICK, M.D. 1897.
*Influenza.* By WILLIAM GRAY, M.D., C.M. 1897.

From J. B. LIPPINCOTT COMPANY, London:

*The Practice of Medicine.* By HORATIO C. WOOD, A.M., M.D., LL.D., and REGINALD H. FITZ, A.M., M.D. 1897.

From PERCY LUND, HUMPHRIES & Co., London:

*The Practical Photographer.* Vol. VIII. No. 86. February, 1897.

From JAMES MACLEHOSE & SONS, Glasgow:

*Medicine in Shakespeare.* By Dr. JOHN MOYES. 1896.

From MACMILLAN AND Co., LIMITED, London:

*System of Medicine.* By Many Writers. Edited by THOMAS CLIFFORD ALLBUTT, M.A., M.D., LL.D., F.R.S. Volume II. 1897.
*Essays and Addresses.* By Sir J. RUSSELL REYNOLDS, Bart., F.R.S., M.D. 1896.

From HORACE MARSHALL & SON, London:

*Travel.* April, 1897.

From MASSON ET CIE, Paris:

*La Revue philanthropique.* Nos. 1, 2. 1897.

From MORTON & BURT, London:

*Sixty-second Annual Report of the British Medical Benevolent Fund, for the year* 1896.

From NEWTON, CHAMBERS & Co., LTD., Sheffield:

*Bacteriological Report on Izal.* By E. KLEIN, M.D., F.R.S. [1892.]
*Izal in its Surgical Aspects.* By WM. BRUCE CLARKE, M.B. 1894. [Reprint.]
*The Action of Izal in Operations on the Eye.* By ROBERT BRUDENELL CARTER. 1895. [Reprint.]
*The Pharmacology of Izal.* By F. W. TUNNICLIFFE, M.D. 1896. [Reprint.]
*Report on the Disinfecting and Antiseptic Properties of Izal.* By SHERIDAN DELÉPINE. [N.D.]
*Extracts from Chapter on Disinfection;* Taken from *The Theory and Practice of Hygiene.* By J. LANE NOTTER, M.A., M.D., and R. H. FIRTH.
*Izal, its History and Chemical Properties.* [N.D.]
*Resumé of Ascertained Actions and Uses of Izal.* [N.D.]

From YOUNG J. PENTLAND, Edinburgh:

*Diseases of the Liver, Gall Bladder, etc.* By H. J. WARING, M.S., B.Sc. 1897.
*Edinburgh Medical Journal.* New Series. Vol. I. 1897.

From JOHN MORGAN RICHARDS, London:

*Medical Reprints.* March—May, 1897.

From RIVINGTON, PERCIVAL & Co., London:

*Infantile Syphilis.* By J. A. COUTTS, M.B. 1897.

From GEORGE R. RYLEY, Halifax:

*The Scalpel.* Vol. II. No. 17. May, 1897.

From W. B. SAUNDERS, Philadelphia (Per the Rebman Publishing Company, Ltd.):

*Pathology and Surgical Treatment of Tumors.* By N. SENN, M.D., Ph.D, LL.D. 1895.

From THE SCIENTIFIC PRESS, LIMITED, London:

*The Matron's Course.* By Miss S. E. ORME. 1897.

From AUG. SIEGLE, London:

*The Therapist.* March, 1897.

From N. SIMMONS, Bristol:

*Simmons' Perfect System of Sanitation.*

From J. W. SIMPSON, Derby:

*Derby Borough Asylum. Eighth Annual Report,* 1896. 1897.

From GEORGE TIEMANN & Co., New York:

*Tiemann's Reprints.* No. 16. [N.D.]

From WILLIAMS AND NORGATE, London.

*Problems of Nature, Researches and Discoveries of Gustav Jaeger, M.D.* Edited and Translated by HENRY G. SCHLICHTER, D.Sc. 1897.

From JOHN WRIGHT & Co., Bristol:

*The Swedish System of Physical Education.* By THEODORA JOHNSON. 1897.

